# Caspase-3 dependent nitrergic neuronal apoptosis following cavernous nerve injury is mediated via RhoA and ROCK activation in major pelvic ganglion

**DOI:** 10.1038/srep29416

**Published:** 2016-07-08

**Authors:** Johanna L. Hannan, Hotaka Matsui, Nikolai A. Sopko, Xiaopu Liu, Emmanuel Weyne, Maarten Albersen, Joseph W. Watson, Ahmet Hoke, Arthur L. Burnett, Trinity J. Bivalacqua

**Affiliations:** 1 Department of Physiology, Brody School of Medicine, East Carolina University, 600 Moye Boulevard, Greenville, NC, 27834, USA; 2 Department of Urology, Doai Memorial Hospital and The University of Tokyo, Tokyo, Japan; 3 The James Buchanan Brady Urological Institute, and Department of Urology, The Johns Hopkins School of Medicine, 600 North Wolfe Street, Marburg 420, Baltimore, MD, 21287, USA; 4 Laboratory of Experimental Urology, Department of Urology, KU Leuven, Leuven, Belgium; 5 Department of Neurology, The Johns Hopkins School of Medicine, Baltimore, MD, 21287, USA

## Abstract

Axonal injury due to prostatectomy leads to Wallerian degeneration of the cavernous nerve (CN) and erectile dysfunction (ED). Return of potency is dependent on axonal regeneration and reinnervation of the penis. Following CN injury (CNI), RhoA and Rho-associated protein kinase (ROCK) increase in penile endothelial and smooth muscle cells. Previous studies indicate that nerve regeneration is hampered by activation of RhoA/ROCK pathway. We evaluated the role of RhoA/ROCK pathway in CN regulation following CNI using a validated rat model. CNI upregulated gene and protein expression of RhoA/ROCK and caspase-3 mediated apoptosis in the major pelvic ganglion (MPG). ROCK inhibitor (ROCK-I) prevented upregulation of RhoA/ROCK pathway as well as activation of caspase-3 in the MPG. Following CNI, there was decrease in the dimer to monomer ratio of neuronal nitric oxide synthase (nNOS) protein and lowered NOS activity in the MPG, which were prevented by ROCK-I. CNI lowered intracavernous pressure and impaired non-adrenergic non-cholinergic-mediated relaxation in the penis, consistent with ED. ROCK-I maintained the intracavernous pressure and non-adrenergic non-cholinergic-mediated relaxation in the penis following CNI. These results suggest that activation of RhoA/ROCK pathway mediates caspase-3 dependent apoptosis of nitrergic neurons in the MPG following CNI and that ROCK-I can prevent post-prostatectomy ED.

Despite nerve-sparing radical prostatectomy for the treatment of clinically localized prostate cancer, there is still a significant amount of men that develop severe erectile dysfunction (ED) post-surgical intervention^1^. The axonal injury which occurs is a classic peripheral neuropathy with an acute inflammatory phase leading to Wallerian degeneration of the autonomic nerves and an ultimate regenerative phase governed by enhanced neurotrophic factor activation and reduction in neuroinflammation^2^. Type 5 phosphodiesterase inhibitors (PDE5I) are utilized to treat post-RP ED^1^; however, PDE5I only affect corporal smooth muscle cell function and do not serve as a neurotrophic agent. Accumulating evidence suggests recovery of erectile function following injury to the cavernous nerves (CN) is dependent, in part, upon axonal regeneration and successful functional reinnervation of the end-organ penis with intact neuronal nitric oxide (NO) release^3^. Therefore, novel targets are sought to assist erection recovery which focus on the protection of both vascular and more importantly, neuronal function.

The RhoA/Rho-associated protein kinase (ROCK) pathway plays an important NO-independent role in regulating smooth muscle tone in penile tissues and has off target effects on NO signaling in the penis^4^,^5^. Increased activity of the RhoA/ROCK pathway is involved in several pathologic disease processes of the penis^6^,^7^,^8^,^9^,^10^. Following peripheral CN injury (CNI), membrane bound RhoA and ROCK increase in the corporal endothelial and smooth muscle cells of the penis^11^,^12^. This is associated with decreased nitrergic and endothelial nitric oxide synthase (NOS; nNOS and eNOS) protein expression and increased corporal tissue apoptosis^4^. The mechanisms hypothesized to control the upregulation of penile RhoA and ROCK are thought to be secondary to denervation of the end-organ penis, increased adrenergic tone, and ischemia resulting from spontaneous tumescence. ROCK inhibition (ROCK-I) utilizing Y-27632 (a synthetic competitive inhibitor specific to ROCK1 and ROCK2) following CNI improves erectile function via mechanisms directly related to restored NO-biosynthesis and decreased apoptosis in the penis^11^,^12^.

Although increased ROCK activity has been ascribed to cause increased penile vascular tone and ED, its role in regulation of autonomic CN function following injury has not been elucidated. In the central nervous system (CNS), RhoA/ROCK mediates growth inhibitory signals generated by myelin-associated inhibitors via growth factor stimulus of RhoA GTPase activity^13^,^14^,^15^. After CNS injury, RhoA and ROCK are activated which leads to cytoskeleton rearrangements of neurons, inhibition of neurite outgrowth and apoptosis^16^. In the peripheral nervous system (PNS), axonomy and injury upregulates transcription and translation of RhoA and ROCK, and pharmacologic inhibition *in vitro* and *in vivo* increases axonal regeneration following peripheral nerve injury^17^,^18^,^19^. Taken together, these studies indicate that peripheral regeneration is hampered by concurrent activation of the regenerative brake RhoA.

The role of the RhoA/ROCK signaling cascade in the major pelvic ganglia (MPG) and the peripheral autonomic CN following injury is unknown. In this study, we demonstrate that upregulation of the RhoA/ROCK pathway in the MPG leads to direct activation of caspase-3 and selective apoptosis of nitrergic neurons. Using a validated pre-clinical model to study the peripheral effects of CNI^11^,^12^, we demonstrate that inhibition of ROCK will prevent apoptosis in the MPG, and restore nitrergic signaling to the penis thus preventing the detrimental effects of axonal degeneration to the penis that occur after CNI.

## Results

### Impact of CNI on MPG RhoA/ROCK and neuronal marker gene and protein expression

**Figure 1:**
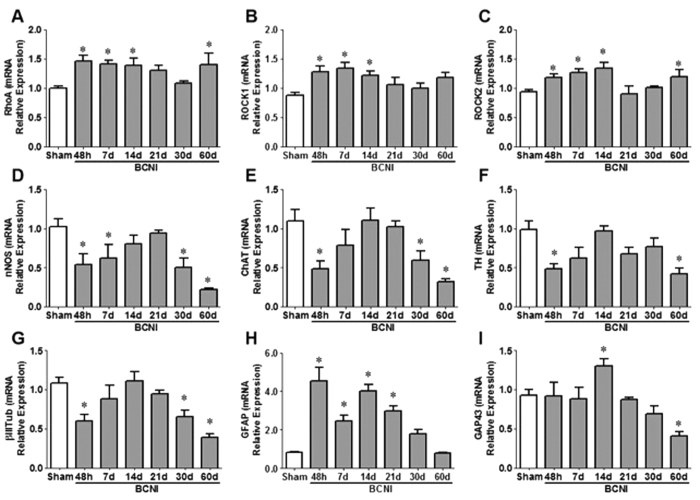
Time course of gene expression of RhoA/ROCK and neuronal markers in the MPG following CNI. The gene expressions of RhoA (**A**), ROCK1 (**B**), ROCK2 (**C**), markers for nitrergic (nNOS, **D**), parasympathetic (ChAT, **E**), sympathetic (TH, **F**) neurons and the neuronal marker βIIITub (**G**), markers for nitrergic (nNOS, **D**), parasympathetic (ChAT, **E**), sympathetic (TH, **F**) neurons, neuronal marker βIIITub (**G**), a marker of Schwann cells (GFAP, **H**) and marker of neuronal growth (GAP43, **I**) were measured. Each bar represents mean ± SEM normalized to the mRNA relative expression of HPRT1 (n = 6–8/group). *P < 0.05 compared to Sham.

**Figure 2:**
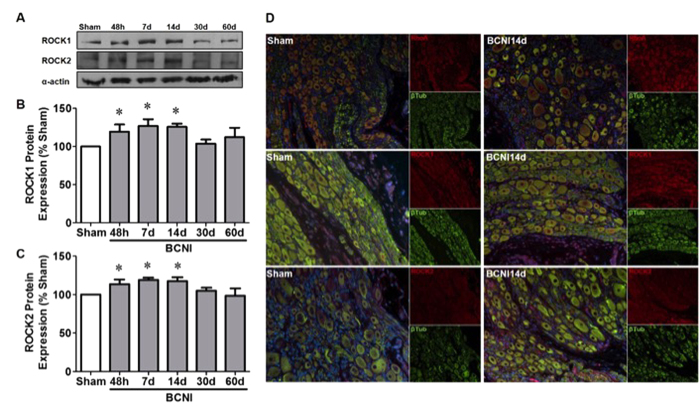
Protein expression and immunofluorescent localization of RhoA/ROCK signaling proteins in the MPG. Representative images for Western blots of ROCK1, ROCK2 and α-actin (**A**). Quantification was performed by densitometry, normalized to α-actin and expressed as a percentage of Sham (**B**,**C**). Each bar represents mean ± SEM (n = 6/group). *P < 0.05 compared to Sham. (**D**) Paraffin-embedded sections were stained with RhoA, ROCK1 or ROCK2 (red), βIII tubulin (green) and nuclear stain DAPI (blue). Magnification 200x.

Early temporal increases in RhoA, ROCK1 and ROCK2 mRNA expressions were evident 48 hours to 14 days after CNI and decreased by 30 days post-CNI (Fig. 1A–C). The gene expressions of markers for nitrergic (nNOS), parasympathetic (ChAT), sympathetic (TH) neurons and the neuronal marker (βIIITub) were decreased by 50% within 48 hours of CNI (Fig. 1D–F,I). Interestingly, the gene expression of these markers increased, peaked at 14–21 days and remained 50% lower than sham at 30–60 days. The mRNA expression of GFAP, a marker of Schwann cells, was markedly increased 48 hours to 21 days after CNI (Fig. 1G). GAP43, a marker of neuronal growth, peaked 14 days subsequent to bilateral CNI and fell below sham levels (Fig. 1H). Increases in the protein expression of ROCK1 and ROCK2 was also evident 48 hours to 14 days post-CNI (Fig. 2A–C). Representative images of immunofluorescence staining demonstrate localization of RhoA in the cavernous nerve as well as the MPG neuronal cell body and nucleolus (Fig. 2D–I). ROCK1 and ROCK2 staining is primarily located in the neuronal cell bodies following CNI.

### CNI increased caspase-3 mediated apoptosis in MPG

**Figure 3:**
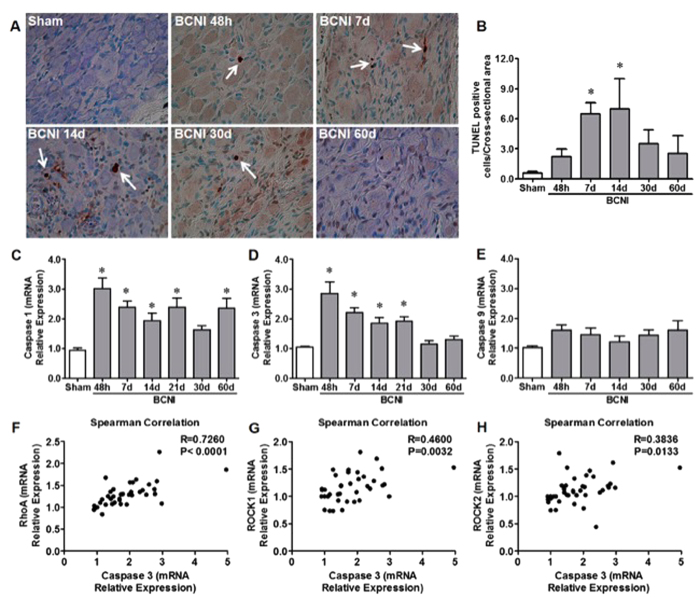
CNI induced an increase in TUNEL positive cells, and caspase-1 and -3 in the MPG that was significantly correlated with the gene expression of RhoA/ROCK. TUNEL positive stained cells (white arrows, **A**) were counted from control and CNI MPGs and normalized to the cross-sectional area of the MPG (**B**; n = 6/group). The mRNA expression of caspase-1 (**C**), -3 (**D**), and -9 (**E**) was normalized to HPRT1 gene expression and bars represent the mean ± SEM. *P < 0.05 compared to Sham. Scatter diagrams demonstrate the correlation between MPG caspase-3 gene expression levels and RhoA (**F**), ROCK1 (**G**) and ROCK2 (**H**) gene expression levels from Sham and all injured time points. R = Spearman correlation coefficient.

The number of TUNEL-positive apoptotic cells was increased in the MPG 7 and 14 days post-CNI (Fig. 3A,B). The increased apoptosis occurred in parallel with increased MPG gene expression of caspase 1 and 3 between 48 hours and 21 days post-CNI (Fig. 3C,D). There was no change in the mRNA expression of caspase 9 (Fig. 3E). A positive correlation in gene expression of RhoA, ROCK1 or ROCK2 to caspase 3 mRNA was observed indicating that an upregulation of RhoA, ROCK1, and ROCK2 correlates with the increase in apoptosis in the MPG after CNI.

### ROCK-I prevents RhoA/ROCK mediated caspase activation

**Figure 4:**
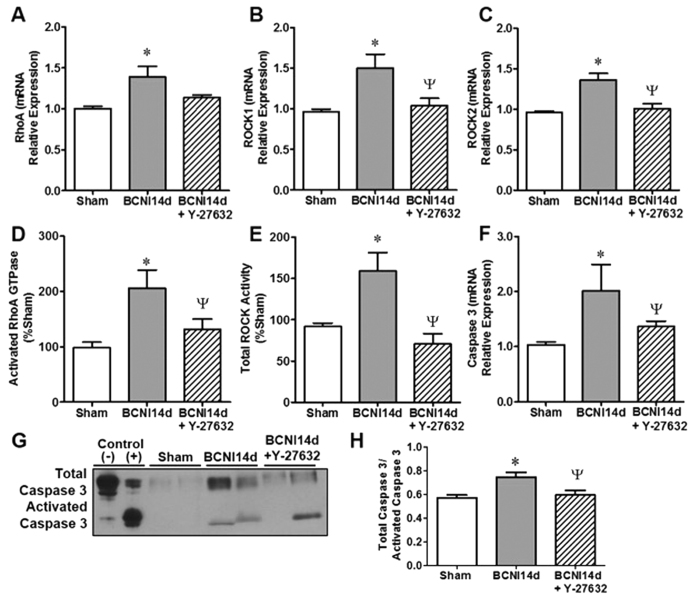
Treatment with Y-27632 lowered the RhoA/ROCK and caspase-3 gene expression and activity of CNI MPGs to sham levels. Gene expression of RhoA (**A**), ROCK1 (**B**) and ROCK2 (**C**) was lowered with ROCK-I. Similarly, ROCK-I prevented elevated RhoA GTPase activity (**D**) and total ROCK activity (**E**) caused by CNI. Elevated gene expression of caspase-3 was also prevented by treatment with a ROCK inhibitor (**F**). Western blot analysis of caspase-3 control cell extracts treated with or without cytochrome c were used to confirm full length and cleaved activated caspase 3 (**G**). Quantification was performed by densitometry and expressed as the ratio of total caspase-3 to activated caspase-3 (**H**). Each bar represents mean ± SEM. Gene expression data were normalized to the mRNA relative expression of HPRT1. *P < 0.05 compared to Sham. ^Ψ^P < 0.05 compared to CNI 14d.

Elevated gene expressions of RhoA, ROCK1 and ROCK2 14 days following CNI were prevented by ROCK-I in the MPG (Fig. 4A–C). Following CNI, the MPG activity of RhoA GTPase and total ROCK activity was 2-fold higher than sham and inhibition of ROCK prevented the increased activity of both RhoA GTPase and total ROCK activity (Fig. 4D,E). The CNI-induced increase in gene expression of caspase 3 was also prevented by treatment with a ROCK inhibitor (Fig. 4F). Western blot analysis of total and activated caspase 3 showed an increase in the ratio of total to activated caspase 3 protein in CNI 14d MPGs which was significantly lower following ROCK inhibition (Fig. 4G,H).

### nNOS uncoupling is prevented with ROCK-I

**Figure 5:**
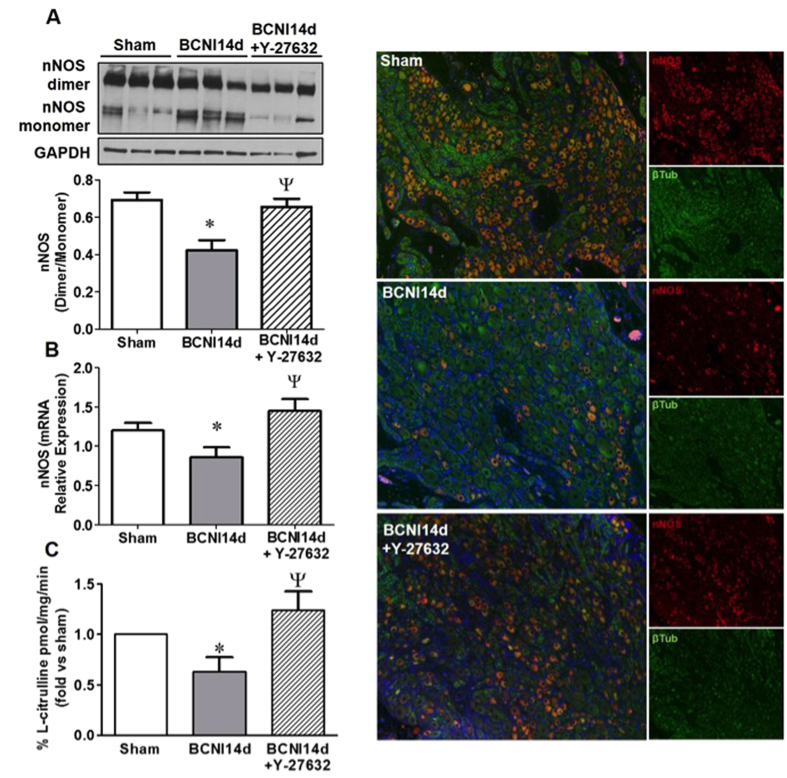
ROCK-I prevented nNOS uncoupling and preserved nNOS gene expression and activity in the MPGs of CNI rats. CNI uncoupled nNOS protein and lead to decreased dimer to monomer ratio (**A**) and lower relative nNOS mRNA expression (**B**). Y-27632 prevented nNOS uncoupling and increased nNOS gene expression. Western blot quantification was performed by densitometry, normalized to GAPDH and gene expression was normalized to the relative expression of HRPT1. Each bar represents mean ± SEM (n = 6/group). *P < 0.05 compared to sham and ^Ψ^P < 0.05 to CN14d. NOS activity assessed by measuring L-citrulline production was lowered in CNI14d MPGs and maintained at sham levels with ROCK inhibition (**C**). Paraffin-embedded sections stained with nNOS (red), βIII tubulin (green) and nuclear stain DAPI (blue) demonstrated a decrease in nNOS positive ganglion cell bodies following CNI which was prevented with ROCK inhibition (**D**). Magnification 200x.

The production of NO occurs when nNOS is in its dimerized form and when uncoupled can lead to diminished levels of NO and increased oxidative stress^20^. Following CNI injury, there is decrease in the dimer to monomer ratio of nNOS protein, decreased mRNA expression and lowered nNOS activity in the MPG (Fig. 5A–C). Additionally, immunofluorescence staining demonstrates a qualitative decrease in nNOS positive ganglionic cell bodies (Fig. 5D–F). Treatment with ROCK-I prevents the uncoupling of nNOS and increases the gene expression of nNOS and the activity of nNOS. There is an increase in the number of nNOS positive ganglionic cells compared to CNI MPG with ROCK-I.

### Increased neurite outgrowth with ROCK inhibition

**Figure 6:**
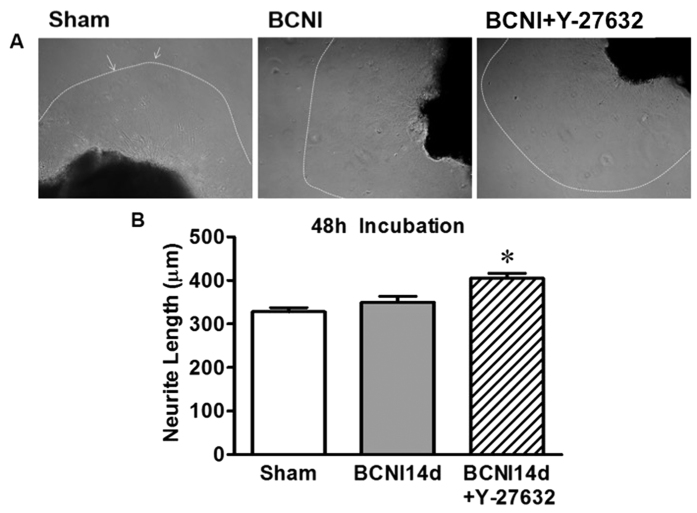
ROCK-I increased neurite outgrowth from the MPG of CNI rats. (**A**) Photomicrograph demonstrating neurite outgrowth from sham, CNI, and CNI + Y-27632 treated rats 14 days post-injury. The MPG were explanted and grown in Matrigel and neurite length was quantified 48 hours after explant. (**B**) Bar graphs demonstrating neurite length (μm) from the three groups of MPG explanted and grown in Matrigel. Each bar represents mean ± SEM (n = 3/group). *P < 0.05 compared to Sham and CNI.

Fourteen days after CNI, sham, CNI, and CNI rodents treated with Y-27632 were sacrificed. The MPGs were then explanted and cultured in Matrigel. Neurite outgrowth in cultured MPGs was measured after 48 hours of incubation. There was no difference in average neurite length in the sham and CNI MPGs (Fig. 6A,B). MPGs cultured in Matrigel from injured rats treated with the ROCK-I demonstrated increased neurite outgrowth in comparison to sham and CNI MPGs (p < 0.05, Fig. 6A,B).

### *In vivo* erectile function and *ex vivo* corporal vasorelaxation is preserved by ROCK-I

**Figure 7:**
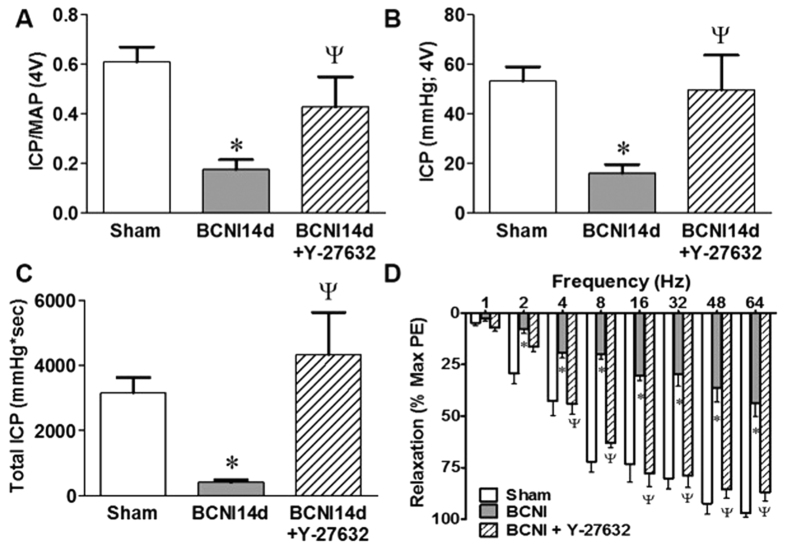
Erectile function and NANC-mediated relaxation was preserved in CNI rats treated with Y-27632. Inhibition of ROCK prevented the decline in ICP/MAP (**A**), maximal ICP (**B**) and total ICP (area under the curve, **C**) after CNI. Additionally, CNI decreased electrical field stimulated NANC-mediated relaxation and ROCK inhibition prevented the decline in relaxation (**D**). Each bar represents mean ± SEM (n = 8/group). *P < 0.05 compared to Sham. ^Ψ^P < 0.05 compared to CNI14d.

As previously shown^21^, CNI leads to significantly lower ICP/MAP, maximal ICP and total ICP (Fig. 7A–C). Further evidence of dysfunctional nitrergic signaling is evident by a decline in the NANC-mediated relaxation in the penis from CNI rats at all frequencies (Fig. 7D). ROCK-I prevented the deficit in erectile function post-CNI and maintained total ICP, ICP/MAP, and total ICP to levels observed in the age-matched sham cohort (Fig. 7A–C). Additionally, NANC-mediated relaxation of corporal tissue at all frequencies tested was also preserved with the inhibition of ROCK (Fig. 7D).

## Discussion

In the present study, the *in vivo* erectile responses and *ex vivo* corporal relaxation results demonstrated that 14 days after bilateral CNI there is a profound decline in nitrergic-nerve mediated erectile responses and corporal vasorelaxation. Identification of alternative pathways contributing to nitrergic axonal degeneration is necessary to advance therapeutic strategies for ED particularly those related to nerve injury and neurological diseases. Thus, we evaluated the activation and expression of RhoA and ROCK in the MPG following CNI in an effort to identify a disease-specific inhibitors of axonal regeneration that contribute to early degeneration of the autonomic innervation of the penis in response to peripheral CNI. The results of the present study demonstrate an early sustained temporal increase in expression of RhoA and ROCK in the MPG after CNI. This increase in gene expression and activation of RhoA and ROCK in the MPG directly correlated with apoptosis in the MPG measured by TUNEL positive cells and caspase 3 gene expression. We also demonstrate that nNOS positive ganglion and gene expression is significantly decreased following bilateral CNI in the MPG. We found that nNOS is uncoupled contributing to the decreased expression and activity of nNOS in the MPG 14 days following bilateral CNI. These data identify RhoA/ROCK as a potential novel signaling pathway activated in response to CNI, which may serve as a regulator of peripheral nitrergic (nNOS) axonal degeneration.

There is a desperate need for understanding evolving mechanisms of neuronal degeneration and regenerative pathways in the MPG and CNs after iatrogenic injury. The animal model chosen for this study was a CN crush injury since this type of peripheral nerve injury is designed to mimic the partial nerve damage that occurs during nerve-sparing radical prostatectomy. The model of bilateral CNI causes a time-dependent decline in neurogenic erectile response to CN stimulation and is the model validated^22^ for studying mechanisms-associated with post-CNI ED (post-prostatectomy ED).

RhoA is a small GTP-binding protein, acting as a molecular switch between the inactive GDP-bound state and the active GTP-bound state^23^. The major downstream effector of RhoA is ROCK1 and ROCK2, which serve as key regulators of penile vascular homeostasis and upon activation cause ED^4^. However, the role in regulation of neuronal autonomic innervation to the penis from the MPG is unknown and identification of inhibitory signaling pathways in the MPG after CNI has not been investigated. Recently, an increase in RhoA and ROCK2 was observed in the MPG in a type 1 diabetic rodent model^24^ suggesting that in a disease state, which is associated with neuropathy, RhoA and ROCK may mediate peripheral autonomic neuropathy of the penis. In the present study, we found a peak in expression and activity of RhoA and both ROCK isoforms in the MPG 14 days post CNI providing evidence that a temporal increase in RhoA and ROCK contributes to the loss of autonomic ganglion cells (nNOS, TH, and ChAT) of the MPG. The localization of RhoA/ROCK expression was found in both the ganglion cell bodies and nerve fibers. These findings implicate that RhoA/ROCK may influence CN function.

An interesting finding from this study was the rapid decrease in gene expression of nNOS, TH, and ChAT with a temporal increase in expression peaking at 14–21 days with another significant decline in expression in the MPG at 30 and 60 days post-injury. This change in expression follows the temporal trend in expression of the house keeping gene β-tubulin III at the same time points. Previously, we have demonstrated that *in vivo* neurogenic erectile responses to CN stimulation following injury have a time-dependent decline in erectile function with the most significant decline at 14 days and improvement in neurogenic-mediated erections up to 60 days post injury^21^. Prior studies have shown the importance of Schwann cell activation in peripheral nerve regeneration^25^,^26^. The duration of their involvement was previously unknown. We demonstrate increased GFAP expression in the MPG for all time points up to 21 days following injury suggesting they are involved in early nerve remodeling. Taken together, the results of the present study and previous investigations suggest that despite improvement in physiologic erections there is genomic changes in autonomic ganglion and neurons which is lower (~50% fold change) than age-matched rat MPG but there is still continued regeneration of nerves which provide adequate innervation to the penis to restore normal penile homeostasis.

Bilateral CNI reduced nNOS expression in the dorsal nerve of the penis as a result of neurodegeneration following neuropraxia^27^. We have previously demonstrated that ROCK-I prevented a decline in nNOS expression in CNI rat penes^11^. The preservation of nNOS levels and neurogenic-mediated erectile responses indicate that the autonomic innervation to the corpora cavernosa is conserved in animals treated with ROCK-I. RhoA/ROCK signaling pathway is activated in neural tissue following nerve injury and ischemia in the central nervous system^13^,^14^,^15^. Activation of ROCK can inhibit axon elongation and result in growth cone collapse in regenerating neurons^15^. Here we show an increase in RhoA, ROCK1 and ROCK2 gene and protein expression as well as RhoA GTPase and total ROCK activity in the MPG 14 days post CNI. Thus, inhibition of ROCK may promote CN regeneration following peripheral crush injury. In the present study, we found a number of novel findings: CNI results in a reduction in nitrergic (nNOS) gene expression, uncoupling of nNOS protein with resultant decline in nNOS enzymatic activity, and decreased axonal staining of nNOS in the MPG 14 days after CNI. Moreover, we show that inhibition of ROCK with Y-27632 prevents the uncoupling of nNOS and increases nNOS gene expression and activity in the MPG. This directly results in preserved neurogenic mediated *in vivo* and *ex vivo* nitrergic relaxation of the corpora cavernosa thus providing direct evidence for RhoA and ROCK inhibitory function on nitrergic axons of the MPG.

Activation of RhoA/ROCK following peripheral nerve injury has been shown to inhibit neurite outgrowth in spinal motor and peripheral sensory neurons^28^. We’ve demonstrated that MPGs cultured in Matrigel from CNI rats treated with Y-27632 have increased neurite sprouting and growth. Explanted MPGs from Y-27632-treated BCNI rats demonstrated decreased RhoA staining, which is consistent with prior studies. In several tissue types, including motor neurons, ROCK-I has been shown to decrease its upstream regulator RhoA^29^,^30^,^31^,^32^. This feedback inhibition may further augment nerve regeneration and contribute to the increased neuritogenesis from the MPG following ROCK inhibition seen in our model. The specific types of neurites (adrenergic, cholinergic, nitrergic), which show increased growth from the MPG, are unknown at this time and will be the focus of future exploratory studies.

The intrinsic capability of adult peripheral neurons to regenerate is accompanied by a non-permissive environment consisting of soluble inhibitory molecules, which counteract a regenerative response and promote apoptosis. Recent evidence demonstrates that activation of RhoA and ROCK in peripheral nerves inhibits neurogenesis after injury^17^ and based on our previous^11^,^12^ and current study we clearly show that activation of RhoA/ROCK occurs in the MPG after CNI and represents a novel signaling pathway that inhibits neurite outgrowth after injury. Here we show that inhibition of ROCK prevents nitrergic degeneration in the MPG, promotes neuritogenesis of MPG neurites *ex vivo* and preserved autonomic innervation to the penis. The mechanism by which this occurs appears to be related to inhibition of caspase-3 dependent apoptosis of ganglia in the MPG. We have previously shown using a whole genomic microarray that pro-apoptotic genes, including caspase-1 and 3, are activated early (48 hours) and 14 days post CNI in the MPG^33^. In the present study, we show that TUNEL-positive apoptotic cells increase in the MPG 7 and 14 days post-CNI with an associated increase in gene expression of both caspase 1 and 3 between 48 hours and 21 days post-injury. We also demonstrated that activated caspase-3 protein expression is increased 14 days after injury. Of particular interest is the observation that a positive correlation in gene expression of RhoA and ROCK to caspase 3 in the MPG which strongly suggesting that the RhoA/ROCK signaling axis mediates the increase in apoptosis in the MPG after CNI. This is further supported by the effect of ROCK-I prevents caspase-3 dependent apoptosis 14 days post-CNI. These results are consistent with previous reports in the CNS by which inhibition of ROCK prevented apoptosis via a capse-3 dependent mechanism^14^,^34^.

Previously, in both the CNS and PNS, the use of ROCK-I (fasudil, Y-27632) enhance axonal regeneration with improvement in motor function^16^. Consistent with the results from our *ex vivo* explanted MPG, we found that daily systemic administration of Y-27632 for 11 days with a three day wash out period to avoid direct vascular effects preserved erectile responses to CN stimulation *in vivo* and *ex vivo* corporal myograph responsiveness to activation of NANC nerves that was similar to age-matched sham rats. The CNI group had significantly impaired end organ penile vascular function consistent with a peripheral neuropathy. Systemic ROCK-I prevented both RhoA GTPase activity and total ROCK activity in the MPG and decreased RhoA and ROCK gene expression 14 days after CNI. We chose the 14 day post-crush time point in our peripheral CNI model for all physiologic, molecular, and biochemical analysis in order to evaluate a time when axonal degeneration and early axonal regeneration is occurring with end organ target re-innervation^21^. Y-27632 is a non-selective ROCK inhibitor and the specific isoform of ROCK responsible for the pathologic effects observed in this study cannot be assessed; however, it’s unlikely that one isoform is more detrimental in this experimental model since both ROCK1 and 2 are increased following CNI.

While *in vivo* and *ex vivo* penile hemodynamics and corporal responsiveness were preserved following daily treatment with the selective ROCK-I, Y-27632, one limitation is that it remains undetermined whether this is a beneficial physiologic effect that can be found after an established injury has occurred to CN and MPG. Moreover, can a delay in treatment with a ROCK-I also promote regeneration of nitrergic axons with reversal of the autonomic neuropathy to the penis? Further studies examining the role of the RhoA/ROCK pathway in the MPG and penile innervation are ongoing in our laboratory to address this question. Also, the upstream activators of RhoA, such as local induction of macrophages and myelin-associated molecules, glycoproteins or chondroitin sulphate proteoglycans (CSPGs) following injury were not addressed in this study and warrant further investigation^35^.

Besides its pertinence to the field of ED, the study of RhoA/ROCK signaling in axonal degeneration of the CN following crush injury carries additional implications with regard to the field of peripheral axonal regeneration. Knowledge gained in the present study of activated RhoA in the neuroregulation of penile erection provides the first scientific evidence of therapeutic inhibitors of ROCK demonstrating benefit on peripheral nerve regeneration via mechanisms involving inhibition of caspase-3 dependent apoptosis and prevention of nitrergic degeneration in the MPG following peripheral CN-injury.

## Conclusions

The results of this study indicate that RhoA/ROCK signaling cascade in the MPG promotes apoptosis and nitrergic dysfunction following CNI. Furthermore, systemic administration of the pharmacologic inhibitor of ROCK (Y-27632) prevents caspase-3 dependent apoptosis of nNOS positive axons, nNOS uncoupling and enzymatic activity in the MPG. Inhibition of ROCK promoted neuritogenesis from explanted MPG and preserved nitrergic nerve mediated erections and corporal relaxation in the penis. Therefore, we believe ROCK-I may be an important target to preserve axonal autonomic innervation to the penis following nerve-sparing radical prostatectomy.

## Materials and Methods

### Experimental design

All experiments were approved by the Johns Hopkins University School of Medicine Animal Care and Use Committee in accordance with the National Institutes of Health Guide for the Care and Use of Laboratory Animals. Male Sprague-Dawley rats (Charles River, Wilmington, MA) weighing 300–325 grams were used and housed in 12 h light/dark lighting cycle with free access to food and water.

The first experiment randomly assigned 128 rats to 7 groups to undergo sham or bilateral cavernous nerve injury (CNI) surgery. Subsequent to surgery, MPG tissue was collected for histological analysis, Western blotting, PCR and activity assays after 48 hours, 7, 14, 21, 30 and 60 days (Table 1).

The second experiment used 96 rats to examine the effects of ROCK-I on CNI. Animals were assigned to sham, CNI or CNI plus twice daily intraperitoneal (IP) injections with ROCK inhibitor Y-27632 (5 mg/kg; Tocris Bioscience, Ellisville, MO, USA). ROCK-I was withdrawn 72 hours prior to erectile function testing and tissue collection 14 days post-CNI (Table 1).

### CNI and sham surgeries

Under anesthesia, the prostate was exposed via a midline laparotomy and CN identified^11^,^12^,^36^. CNI was induced by applying forceps for 15 seconds x 3 to the nerve 2–3 mm distal to the MPG^37^. In sham animals the CN were identified and the abdomen closed.

### Quantitative PCR (qPCR)

MPGs from sham, 48 hour, 7, 14, 21, 30, 60 day rats were homogenized and total RNA was extracted and purified using the RNeasy system (Qiagen, Hilden, Germany), quantified and then reverse transcribed (GE Healthcore, Pittsburgh, PA, USA)^21^. Real-time qPCR was performed using the StepOnePlus system (Applied Biosystems, Foster City, CA, USA). TaqMan gene expression assays for RhoA, ROCK1, ROCK2, nNOS, choline ester transferase (ChAT), tyrosine hydroxylase (TH), type 3 beta-tubulin (βIIITub), glial factor activating protein (GFAP), growth associated protein 43 (GAP43), caspase-1, caspase-3, caspase-9, activating transcription factor 3 (ATF3) and hypoxanthine phosphoribosyltransferase 1 (HPRT1) were used (Applied Biosystems). HPRT1 was unchanged and served as an endogenous control. All experiments were performed in triplicate (n = 8/group).

### Western blotting

MPGs were excised and homogenized in a Tris-HCL (pH 7.5) buffer to evaluate the protein level of ROCK1 and ROCK2 by Western blot^12^. Sol-O-Buffer (Fabgenix, Frisco, TX) was used to assay nNOS monomers and dimers in the MPG under non-reducing conditions^38^. Protein (30 μg) was loaded on a 4–12% NuPage Bis-Tris gel (Invitrogen, Carlbad, CA) and separated by SDS-polyacrylamide gel electrophoresis (SDS-PAGE). Electrophoresis was done at 4 °C for nNOS monomers and dimers. Proteins were transferred to polyvinylidene fluoride membranes and incubated with primary antibodies (ROCK1, ROCK2 (1:1000, Santa Cruz Biotechnology, Santa Cruz, CA), total casapse-3, activated caspase-3 (1:1000 Cell Signaling, Beverly, MA), nNOS (1:200 Santa Cruz), GAPDH (1:2500 BD Bioscience) and actin (1:500 (Sigma Aldrich, St. Louis, MO)) overnight at 4 °C. The membranes were incubated with a horseradish peroxidase-linked secondary antibody and visualized using an enhanced chemiluminescence kit (Amersham). The densitometry results were quantified using software Image J (National Institutes of Health) and normalized by actin level^37^.

### Histologic analysis

The same animals were used for histological analysis and MPG culture with one MPG going to each experimental procedure. MPGs were formalin fixed and immunofluorescence staining was performed as previously described^37^. Primary antibodies assessed were RhoA, ROCK1, ROCK2 (1:400, Santa Cruz Biotechlogy, Santa Cruz, CA), nNOS (1:50, BD Bioscience, Franklin Lakes, NJ), βIIITub (1:500, Abcam, Cambridge, UK). Secondary antibodies were Alexa-488- and Alexa-594-conjugated (1:200; Invitrogen) and nuclei were stained with 4′,6-diamidino-2-phenylindole (DAPI; Invitrogen). A separate set of slides were stained for TUNEL as per manufacturer’s instructions (ApopTag Peroxidase *In Situ* Apoptosis Detection Kit, Millipore) and counter stained with Hematoxylin (Vector Laboratories, Burlingame, CA). The counting of apoptotic cells was performed by 2 blinded individuals and normalized to the total area of the MPG.

### Total ROCK, RhoA GTPase and nNOS activity assays

A separate group of MPGs were homogenized and total ROCK activity was analyzed in MPG lysate with of 0.1 mM adenosine triphosphate by a ROCK activity assay kit using peroxidase coupled anti-phospho-MYPT1 Thr696 monoclonal antibody (CyClex, Nagano, Japan). RhoA GTPase activity was assayed in MPG lysates by 96-well activity assays (Cytoskeleton, Denver, Colorado) according to manufacturer specifications. Total ROCK and RhoA GTPase activity are displayed as a percent of sham activity, as previously described^8^,^12^.

NOS activity in MPG tissues was assessed by radiolabeled L-arginine to L-citrulline conversion as previously described (Cayman, Chemical, Ann Arbor, MI, USA)^8^,^39^. Constitutive NOS activity measurements were performed in the presence of calcium and shown as a fold change from sham MPG values.

### MPG Culture and Neurite Outgrowth Assessment

Whole MPGs were excised and embedded on reduced growth factor Matrigel in serum free media (RPMI 1640 with 1% Penicillin-Streptomycin, GIBCO) as described (n = 3/group)^21^. All MPGs were covered with 1 ml of media with VEGF (vascular endothelial growth factor; 25 μg/ml, R&D Systems, Minneapolis, MN, USA) which was changed every 24 hours and maintained at 37 °C in a humidified atmosphere with 5% CO_2_. Photographs of neurite growth were captured 48 h following implantation using a Nikon TE200 inverted microscope attached to a CCD Camera and digital images were analyzed with Elements software (Nikon Instruments, Melville, NY, USA). In each area of growth from the MPG the 5 longest neurites were measured^21^. The averages of these measurements defined the neurite length for each groups (20–25 neurites measured per MPG).

### Erectile function measurement

Under anesthesia, the left crus was cannulated with a 25 G needle connected to a pressure transducer to measure intracavernous pressure (ICP)^37^. The right carotid artery was cannulated for continuous measurement of mean arterial pressure (MAP). The CN distal to the crush injury was stimulated with a square pulse stimulator (Grass Instruments, Quincy, MA, USA) at a frequency of 20 Hz, 0.5 msec duration, pulse width of 30 seconds at 4 volts for one minute. Outcome parameters used were ICP/MAP, peak ICP, and total ICP (area under the curve, AUC)^21^.

### Myograph tissue bath experiments

The penis was cleaned of the urethra, dorsal vessels and nerves, cut in 2 × 10 mm strips and mounted in a muscle strip myograph (DMT, Denmark)^40^. Strips were placed in a physiological salt solution, bubbled with 95% O_2_/5% CO_2_ at 37 °C, stretched 4–5 mN, and equilibrated for an hour^41^. Non-adrenergic, non-cholinergic (NANC) mediated relaxation was assessed by electrical field stimulation (EFS) in penile strips incubated with atropine (3 × 10^−5^ M, Sigma) and guanethidine (10^−5^ M, Sigma) for 30 mins. Strips were precontracted with phenylephrine (10^−5^ M) and stimulated at 40 V, 3-ms pulse width at increasing frequencies (1–64 Hz) for 10 sec with 2 min between stimuli. EFS responses were measured as percent relaxation from the pre-contraction to phenylephrine.

### Statistical Analysis

Data were expressed as mean ± SEM. Differences between multiple groups were compared by analysis of variance (ANOVA) followed by a Tukey’s multiple comparisons test. Two-group analysis was performed by unpaired *t*-test. P values of less than 0.05 were used as criteria for statistical significance.

## Additional Information

**How to cite this article**: Hannan, J. L. *et al*. Caspase-3 dependent nitrergic neuronal apoptosis following cavernous nerve injury is mediated via RhoA and ROCK activation in major pelvic ganglion. *Sci. Rep*. **6**, 29416; doi: 10.1038/srep29416 (2016).

## Figures and Tables

**Table 1:** Treatment and control rodent groups used in experiments.

Experiment 1						
Groups	qPCR (n = 56)	Western blots (n = 42)	Histology (n = 30)		Total (n = 128)	
Sham	8	7	5		20	
BCNI 48 h	8	7	5		20	
BCNI 7d	8	7	5		20	
BCNI 14d	8	7	5		20	
BCNI 21d	8				8	
BCNI 30d	8	7	5		20	
BCNI 60d	8	7	5		20	
**Experiment 2**						
**Groups**	**qPCR (n** = **24)**	**Western blots (n** = **21)**	**Histology & Matrigel (n** = **15)**	**Activated RhoA GTPase (n** = **18)**	**Total ROCK Activity (n** = **18)**	**Total (n** = **96)**
Sham	8	7	5	6	6	32
BCNI 14d	8	7	5	6	6	32
BCNI 14d +Y-27632	8	7	5	6	6	32
